# Correlation between the Arrhenius crossover and the glass forming ability in metallic glasses

**DOI:** 10.1038/s41598-017-13611-w

**Published:** 2017-10-13

**Authors:** Tongqi Wen, Wenjing Yao, Nan Wang

**Affiliations:** MOE Key Laboratory of Materials Physics and Chemistry under Extraordinary Conditions, School of Natural and Applied Sciences, Northwestern Polytechnical University, Xi’an, 710072 China

## Abstract

The distinctive characteristic of the metallic glass-forming system is that the variation in viscosity with temperature obeys Vogel-Fulcher-Tammann (VFT) relationship in the undercooled state and Arrhenius relationship in the high temperature region. A dimensionless index has thus been proposed based on the Arrhenius-VFT crossover and the classical nucleation rate and growth rate theory to evaluate the glass-forming ability (GFA). The indicator *G*(*a*) is expressed with the combination of *T*_*g*_, the glass transition temperature, *T*_*x*_, the onset crystallization temperature, *T*_*l*_, the liquidus temperature, *T*_0_, the VFT temperature, and *a* a constant that could be determined according to the best correlation between *G*(*a*) and the critical cooling rate (*R*_*c*_). Compared with other GFA indexes, *G*(*a*) shows the best fit with *R*_*c*_, with the square of the correlation coefficient (*R* ^2^) being 0.9238 when *a* = 0.15 for the 23 various alloy systems concerned about. Our results indicate the crossover in the viscosity variation has key effect on GFA and one can use the index *G*(*a*) to predict *R*_*c*_ and GFA for different alloys effectively.

## Introduction

**Figure 1:**
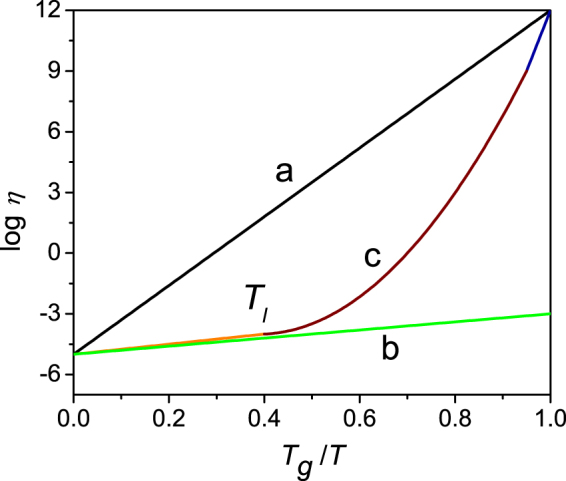
Schematic illustration of the relationships between viscosity (*η*) and temperature with Arrhenius and VFT law. Lines (**a**) and (**b**) denote the Arrhenius relationship in the strong liquids and metallic systems which cannot form glass phase easily, respectively. Line (**c**) demonstrates the VFT relationship for the fragile systems. *T*_*l*_ is the liquidus temperature and *T*_*g*_ is the glass-forming temperature.

Viscosity characterizes the relaxation time of the atoms or molecules in a liquid, and its magnitude plays a key role in the formation of glass phase. Different materials have different viscosity-temperature relationships, as Fig. 1 shows. For a strong liquid system which is a natural glass such as SiO_2_ and GeO_2_, the relationship between the viscosity and temperature (*η*-*T*, with *η* the viscosity and *T* temperature) obeys the Arrhenius relationship (line a), and the viscosity increases strongly with the decrease of temperature. For a metallic system which cannot form glass, *η*-*T* also obeys the Arrhenius relationship (line b). However, the relaxation time of the atom in this kind of materials is short and the viscosity increases slowly with the decreasing temperature. For the metallic glass-forming systems, the investigations in recent years have revealed that *η*-*T* obeys the Vogel-Fulcher-Tammann (VFT)-type relationship^1^:

1 $$\eta ={\eta }_{0}\exp (\frac{{D}_{f}{T}_{0}}{T-{T}_{0}}),$$

as line c in Fig. 1 gives. In the above equation, *η*_0_ is the pre-exponential constant, *D*_*f*_ the fragility parameter, and *T*_0_ is the VFT temperature. At temperature above the liquidus *T*_*l*_, the viscosity still behaves in an Arrhenius-like way. However, it increases sharply with undercooling and obeys the VFT relationship with deviations from the linear Arrhenius. As the temperature decreases and comes close to the glass-forming temperature, the viscosity obeys the Arrhenius relationship again^2^. This slope change, or Arrhenius-VFT (non-Arrhenius) crossover, has aroused much research interest in recent years^3–6^.

Since the increase of viscosity leads to the decrease of diffusivity, and the smaller diffusivity results in shorter characteristic solute diffusion length (*D*/*V*, *D* is diffusion coefficient and *V* is the growth velocity of crystals), it will cause the solute diffusion length to nano scale, which is a necessary condition to obtain glass in metallic system. This raises the question that how to consider the crossover from Arrhenius-VFT in viscosity in judging GFA in metallic systems.

The primary GFA indexes do not consider the contribution of the crossover, such as ${T}_{rg}={T}_{g}/{T}_{l}$ and $\gamma ={T}_{x}/({T}_{g}+{T}_{l})$ ^7,8^, where *T*_*g*_ and *T*_*x*_ are the glass transition temperature and the onset crystallization temperature, respectively. Since they only concentrate on the kinetic critical temperatures and thermodynamic stability, the square of the correlation, *R* ^2^, of these parameters with the critical cooling rate (*R*_*c*_) for many alloy systems is relatively small and not desirable. Later, the slope of the *η*-*T* curve was introduced into indexes. Oleg N. Senkov^9^ proposed an indicator: ${F}_{1}=2\,{[m/{m}_{\min }(1/{T}_{rg}-1)+2]}^{-1}$. Here $m=d{\mathrm{log}}_{10}\tau /d{({T}_{g}/T)}_{T={T}_{g}}$ is the fragility index which determines whether a liquid is strong or fragile, *m*_*min*_ is the minimum fragility index value approximating to 16 for metallic glass, and *τ* is the relaxation time or viscosity. This parameter combines the kinetic critical indication *T*_*rg*_ and fragility index *m* from VFT relation. On one hand, with higher *T*_*rg*_, the nucleation frequency is restrained which stimulates the formation of pure glass phase. On the other hand, a smaller fragility index *m* denotes the characteristics of a stronger liquid. Long *et al*.^10^ found out a new index, $\omega ={T}_{g}/{T}_{x}-2{T}_{g}/({T}_{g}+{T}_{l})$, in which the term $2{T}_{g}/({T}_{g}+{T}_{l})$ denotes the relaxation time that varies proportionally with viscosity at the nose of TTT (time-temperature-transition) curve, and the application of these two parameters shows that they have better correlation with GFA for various alloy systems than *T*_*rg*_ and *γ*. The improvement of the indexes indicates that except for the kinetic process, temperature-related viscosity also contributes to the glassy phase transformation. Takeuchi *et al*.^11^ proposed a new parameter ${\rm{\Delta }}{T}_{g-0}/({T}_{l}-{T}_{0})$ from a creative plot named ${\rm{\Delta }}{T}_{g-0}-scaled$ (${\rm{\Delta }}{T}_{g-0}={T}_{g}-{T}_{0}$) VFT plot. The new parameter $({T}_{g}-{T}_{0})/({T}_{l}-{T}_{0})$ is proposed as only a derivative from the new VFT plot for viscosity and an analog to *T*_*rg*_ with its physical meaning probably to be explained from the aspect of VFT-type viscosity. Based on the discussions on the above parameters, it reveals that the temperature-dependent viscosity, especially the slope change at the Arrhenius-VFT crossover, has a significant influence on *R*_*c*_ and GFA. A large slope at high temperatures and a small slope at low temperatures make the curve approach to the shape of the strong liquids and will be beneficial to form glass phase as can be seen in Fig. 1. In this study, we will consider this point and correlate GFA and the effect of crossover. The correlation is performed firstly by considering the relationship between *R*_*c*_ and the nucleation rate and growth rate^12–14^, then the parameter is proposed by connecting the slope change in the viscosity curve with *R*_*c*_. The derivation process is displayed in the “Method” section. The decent correlation between the parameter and *R*_*c*_ in various glass-forming systems proves the validity of starting from the classical theories. Finally, the new index is compared with some parameters proposed before and the result turns out that it has the best correlation (*R* ^2^ = 0.9238) with *R*_*c*_ among them, validating our indicator is more reliable to characterize GFA.

## Results and Discussions

Now, the index *G*(*a*) derived in the “Method” section is applied to different alloys to verify its validity. The data of 23 glass-forming alloys including bulk metallic glass (BMG), like vitreloy, etc. and marginal glass-forming systems (*R*_*c*_ is more than 10^3^ K/s) is collected, as given in Table 1, for their parameters used in equation (15) can be found in literatures.

**Table 1:** Critical temperatures and the critical cooling rate *R*_*c*_ as well as the value of some parameters proposed before and *G*(0.15).

Alloy	Composition	*R*_*c*_ [K/s]	*T*_*g*_ [K]	*T*_0_ [K]	*T*_*l*_ [K]	*T*_*x*_ [K]	*F*_1_	*ω*	*G*(0.15)
1	Pd_40_Cu_30_Ni_10_P_20_^a^	0.1	577	447	847	657.6	0.4906	0.0670	1.2176
2	Pd_40_Ni_40_P_20_^a^	0.9	582.8	373	964.8	671.7	0.5235	0.1145	1.0835
3	Zr_41.2_Ti_13.8_Cu_12.5_Ni_10_Be_22.5_^a^	1.3	624	412.5	994.5	688.5	0.5331	0.1352	1.1177
4	La_55_Al_25_Ni_5_Cu_10_Co_5_^a^	13.4	456.8	241.2	822.8	541.8	0.5409	0.1291	1.0424
5	La_55_Al_25_Ni_10_Cu_10_^a^	16.3	458.3	254.7	835	547.2	0.5195	0.1288	1.0129
6	Zr_46.75_Ti_8.25_Cu_7.5_Ni_10_Be_27.5_^a^	18	606	372	1102.5	727	0.4852	0.1242	0.9712
7	La_55_Al_25_Ni_15_Cu_5_^a^	27.3	465	273.1	899.8	541.2	0.4688	0.1778	0.9049
8	La_55_Al_25_Ni_5_Cu_15_^a^	39.5	451.2	285.6	878.1	520	0.4369	0.1888	0.8525
9	Mg_65_Cu_25_Y_10_^a^	50	413.7	260	748	478.3	0.4790	0.1527	0.9512
10	La_55_Al_25_Ni_20_^a^	78.3	484	306.5	941.2	555.1	0.4371	0.1927	0.8516
11	Cu_47_Ti_34_Zr_11_Ni_8_^a^	250	680.8	500	1152.4	722.1	0.4340	0.2001	0.8955
12	Pd_77.5_Cu_6_Si_16.5_^a^	300	645	553	1058	682	0.3082	0.1883	0.6846
13	Cu_64_Zr_36_^b^	432	787	649	1230	833	0.3839	0.1644	0.8783
14	Ni_62.4_Nb_37.6_^c^	1400	945	810	1535	923	0.3140	0.2617	0.6811
15	Pd_82_Si_18_^a^	1800	644.6	557	1071	649.6	0.2912	0.2408	0.6296
16	Pt_60_Ni_15_P_25_^d^	4000	500	405	875	500	0.3363	0.2727	0.6876
17	Fe_80_P_13_C_7_^d^	28000	736	616	1258	736	0.3150	0.2618	0.6591
18	Ni_75_Si_8_B_17_^d^	110000	782	670	1340	782	0.2864	0.2630	0.6026
19	Fe_79_Si_10_B_11_^d^	180000	818	701	1419	818	0.2802	0.2687	0.5851
20	Fe_41.5_Ni_41.5_B_17_^d^	350000	720	601	1352	720	0.2736	0.3050	0.5462
21	Co_75_Si_15_B_10_^d^	350000	785	675	1393	785	0.2657	0.2792	0.5481
22	Au_77.8_Ge_13.8_Si_8.4_^e^	740000	294	241.3	629	297	0.2393	0.3528	0.4640
23	Fe_83_B_17_^f^	915000	760	638	1448	760	0.2618	0.3116	0.5207

^a^Refs^9–11,15–18^. (Different values are averaged to get reasonable data).

^b^Refs^10,21^.

^c^Refs^11,22^.

^d^Refs^11,17^.

^e^Refs^11,18^.

^f^Refs^11,17,18^.

**Figure 2:**
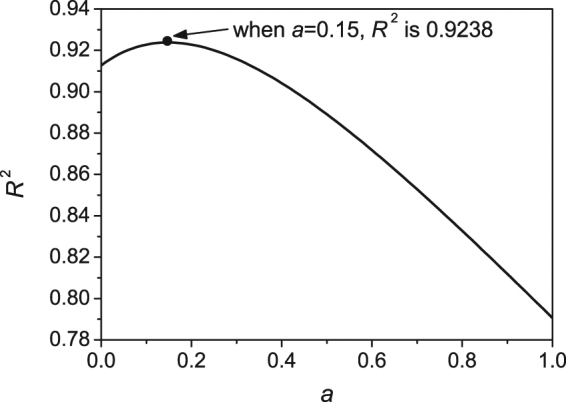
The relationship between *R* ^2^ and parameter *a* for *G*(*a*). Each parameter *a* corresponds to a certain *R* ^2^. *G*(*a*) is the index for glass-forming ability and *a* is a parameter needed to be determined. *R* ^2^ is the square of the correlation coefficient between *G*(*a*) and the critical cooling rate *R*_*c*_.

The parameter *a* is determined according to the best fit between *R*_*c*_ and *G*(*a*) and the relationship between various *a* and *R* ^2^ is then given in Fig. 2. Each *a* corresponds to a certain *R* ^2^ and the maximum of *R* ^2^ is 0.9238 at *a* = 0.15. When *a* = 0, i.e. only considering the influence of *m*_*Tl*_ on GFA, *R* ^2^ = 0.9128. This means compared to the “critical temperature item”, the influence of “viscosity item” on GFA is much more significant.

**Figure 3:**
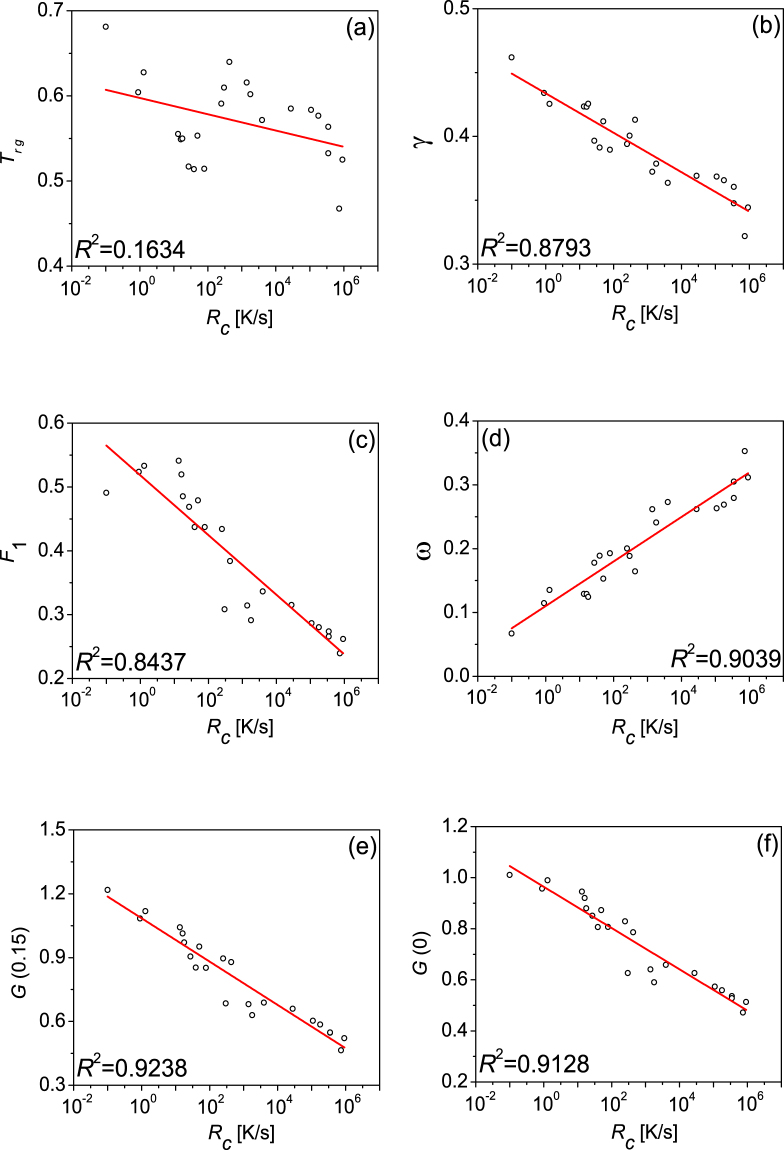
The relationships between different indexes and the critical cooling rates *R*_*c*_. (**a**) ${T}_{rg}={T}_{g}/{T}_{l}$, (**b**) $\gamma ={T}_{x}/({T}_{g}+{T}_{l})$, (**c**) ${F}_{1}=2\,{[m/{m}_{\min }(1/{T}_{rg}-1)+2]}^{-1}$, (**d**) $\omega ={T}_{g}/{T}_{x}-2{T}_{g}/({T}_{g}+{T}_{l})$, (**e**) $G(0.15)=({T}_{g}-{T}_{0}){T}_{l}^{2}/({T}_{g}{({T}_{l}-{T}_{0})}^{2})\cdot {({T}_{x}/({T}_{l}-{T}_{x}))}^{0.15}$, and (**f**) $G(0)=({T}_{g}-{T}_{0}){T}_{l}^{2}/({T}_{g}{({T}_{l}-{T}_{0})}^{2})$.

By using the value of *a* = 0.15 and *a* = 0 respectively, *G*(0.15) and *G*(0) are calculated as functions of *R*_*c*_ and shown in Fig. 3, in which the variations in *T*_*rg*_, *γ*, *F*_1_, and *ω* are also presented for comparison. Data is from the 23 various alloy systems and *R* ^2^ is decided by statistical analysis. Among these GFA indicators, *G*(0.15) has the best correlation with *R*_*c*_, and their relationship can be expressed as:

2 $$G(0.15)=1.08495-0.10185\times \,\mathrm{log}({R}_{c}),$$

This equation could be used to predict *R*_*c*_ for the 23 various metallic glass systems and the data of more systems are needed to further validate the equation.

Now, the mechanisms for the better correlation of *G*(*a*) than other parameters will be discussed. Both critical temperatures and high viscosity are key factors to influence GFA. For *G*(*a*), it considers the contribution of the Arrhenius-VFT crossover in viscosity curve. Other parameters, however, consider little about it. When concentrating on the particular viscosity at a certain temperature, from equations (1) and (10), we have:

3 $$\mathrm{log}(\frac{\eta }{{\eta }_{0}})={m}_{VFT}\frac{{T}_{g}-{T}_{0}}{T-{T}_{0}},$$

Therefore, the relationship between *η*_*l*_ and *T*_*l*_ could be obtained as:

4 $${\eta }_{l}\propto \exp (\frac{{T}_{g}-{T}_{0}}{{T}_{l}-{T}_{0}}),$$

From the basic physical meaning of the equation, considering the starting approximate Arrhenius relationship in Fig. 1, larger viscosity at *T*_*l*_ indicates bigger *m*_*Tl*_ because the viscosity at very high temperature is approximately the same as about 10^−5^ Pa s for many liquids. In this light, larger *η*_*l*_ leads to bigger *m*_*Tl*_ which is related firmly with better GFA.

**Figure 4:**
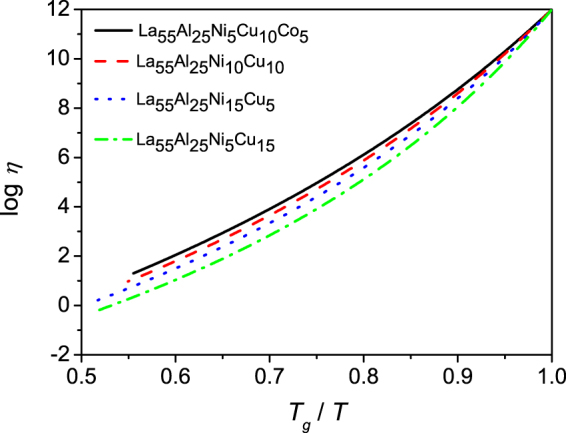
The relationship between viscosity (*η*) and the reduced temperature (*T*_*g*_/*T*) from *T* = *T*_*l*_ to *T* = *T*_*g*_ for four La-based metallic glasses calculated by equation (3).

According to the schematic Fig. 1, GFA is proportional to the viscosity at the liquidus temperature. For showing this effect, the viscosities of four different La-based metallic glasses are calculated by using equation (3), as shown in Fig. 4. For La_55_Al_25_Ni_5_Cu_10_Co_5_, La_55_Al_25_Ni_10_Cu_10_, La_55_Al_25_Ni_15_Cu_5_, and La_55_Al_25_Ni_5_Cu_15_, the sequence of which is according to the viscosity at the liquidus temperature from large to small, *R*_*c*_ is 13.4, 16.3, 27.3 and 39.5 K/s^9–11,15–18^ respectively. Larger *η*_*l*_ suggests bigger $({T}_{g}-{T}_{0})/({T}_{l}-{T}_{0})$ which is also an indication for bigger *m*_*Tl*_ and smaller *m*_*Tg*_. This inverse relationship between *η*_*l*_ and *R*_*c*_ indicates that our indicator is reliable. In this sense, temperature-dependent viscosity is crucial for determining GFA. When considering the classical nucleation rate and growth rate equations (5) and (6), we find that compared to nucleation rate *I*, growth rate *U* is much more dependent on the temperature-dependent viscosity because the value of its square brackets is in the range from 0 to 1. From this perspective, what contribute more to the glass formation are the sluggish diffusion (high viscosity) and the resulting low growth rate. The nucleation rate could be high but the nuclei could not grow because of the nano-scale diffusion length.

## Conclusions

From the above analysis, it can be concluded that a new GFA indicator *G*(*a*) for BMG and marginal metallic glasses is proposed based on the Arrhenius-VFT crossover at *T*_*l*_ as well as the classical nucleation rate and growth rate theory. This index is proved to have better correlation with *R*_*c*_ and GFA for various alloy systems than other parameters proposed before. Furthermore, the calculated results also validate the dependability of using the classical theories mentioned before as the foundation of finding out a new indicator for GFA. Meanwhile, from the analysis result, for simplicity the attention could be paid on the “viscosity item” *m*_*Tl*_, which is the slope at the crossover temperature. This parameter reveals that the temperature dependent viscosity, especially the crossover at *T*_*l*_ and the corresponding viscosity, are crucial for GFA. This could be guidance for developing new glass-forming systems. To be specific, researchers could measure the viscosity at *T*_*l*_, which is applicable because the temperature is relatively high. They could choose the systems with high viscosity at *T*_*l*_ and try to synthesize bulk glass in it. In this sense, the parameter is a theoretical guidance for fabricating new glass-forming systems and could save lots of unnecessary efforts.

## Methods

To correlate the viscosity with the nucleation and growth theory, the following equations for the homogeneous nucleation rate *I* and growth rate *U* are used^12–14^:

5 $$I=\frac{{10}^{35}}{\eta (T)}\exp \,[\frac{-16\pi }{3}\cdot \frac{\Delta {S}_{f}{\alpha }_{m}^{3}{T}^{2}}{{N}_{A}k{({T}_{l}-T)}^{2}}],$$

6 $$U=\frac{kT}{3\pi {a}_{0}^{2}\eta (T)}\,[1-\exp (-\frac{({T}_{l}-T)\Delta {S}_{f}}{{R}_{g}T})],$$

where $k,{\alpha }_{m},{a}_{0},{N}_{A},{\rm{\Delta }}{S}_{f},$ and *R*_*g*_ are Boltzmann constant, a constant of 0.86, mean atomic diameter, Avogadro’s number, the molar fusion entropy, and the gas constant, respectively.

From the amorphous perspective, the crystalline phase has been suppressed until the glass-forming temperature reaches. Therefore, the fraction of the crystallized volume fraction *f*_*c*_ is usually set to be less than 10^−6^. As a result, *R*_*c*_ required for glass formation is determined as^19,20^:

7 $${R}_{c}={[\frac{4\pi }{3\times {10}^{-6}}{\int }_{{T}_{l}}^{{T}_{g}}I(T\text{'}){[{\int }_{T\text{'}}^{{T}_{g}}U(T\text{'}\text{'})dT\text{'}\text{'}]}^{3}dT\text{'}]}^{\frac{1}{4}},$$

Now we will consider the effect of Arrhenius-VFT crossover, as shown in line c in Fig. 1, on the “viscosity item”, $1/\eta (T)$ in equations (5) and (6). Although different values of the crossover temperature were proposed, here we adopt *T*_*l*_ and believe the undercooling starts to contribute to the slope change at this temperature. In this case, for an easy glass-forming system, one expects that the slope of its viscosity at high temperature near *T*_*l*_ is large whereas that at low temperature near *T*_*g*_ is small, so that the system becomes stronger. For this reason, we will determine GFA by combining the slope at the Arrhenius-VFT crossover temperature, *m*_*Tl*_, with that at *T*_*g*_, *m*_*Tg*_.

The slope of the viscosity at a certain temperature *T* can be derived as ${m}_{T}=d(\mathrm{log}\,\eta )/{d({T}_{g}/T)|}_{T}$, thus combine with equation (1) and carry out some simple derivative operations we have

8 $${m}_{{T}_{g}}=\frac{{D}_{f}{T}_{0}{T}_{g}}{{({T}_{g}-{T}_{0})}^{2}\,{\rm{l}}{\rm{n}}\,10},$$

and

9 $${m}_{{T}_{l}}=\frac{{D}_{f}{T}_{0}{{T}_{l}}^{2}}{{T}_{g}{({T}_{l}-{T}_{0})}^{2}\,\mathrm{ln}\,10},$$

According to the theories of Senkov^9^ and Takeuchi^11^,

10 $$\frac{{D}_{f}{T}_{0}}{{T}_{g}-{T}_{0}}={m}_{VFT}\,\mathrm{ln}\,10,$$

where *m*_VFT_ is about 16. Equations (8) and (9) can then be read

11 $${m}_{{T}_{g}}=\frac{{m}_{VFT}{T}_{g}}{{T}_{g}-{T}_{0}},$$

12 $${m}_{{T}_{l}}=\frac{{m}_{VFT}({T}_{g}-{T}_{0}){{T}_{l}}^{2}}{{T}_{g}{({T}_{l}-{T}_{0})}^{2}},$$

Apparently, smaller *m*_*Tg*_ and larger *m*_*Tl*_ determine a stronger liquid and these two slopes are in inverse relationship with each other. Hence, the relationship between GFA and the contribution of viscosity can be given as follows:

13 $${\rm{GFA}}\propto \frac{({T}_{g}-{T}_{0}){{T}_{l}}^{2}}{{T}_{g}{({T}_{l}-{T}_{0})}^{2}},$$

Here *m*_*Tl*_ is used to be included in our GFA index instead of the traditional *m*_*Tg*_ because of its poor correlation with *R*_*c*_^9^. It indicates that the critical temperatures *T*_*g*_, *T*_0_ and *T*_*l*_ play important roles in determining viscosity which is connected firmly with GFA.

For the critical temperature part, we consider the case at *T* = *T*_*x*_ because the nucleation rate at the onset crystallization temperature is decisive for determining GFA. It is known GFA of an alloy melt is proportional to the reciprocals of *I* and *U*, so the relationship between GFA and the “critical temperature item” inside the square brackets could be expressed as:

14 $${\rm{GFA}}\propto \frac{{T}_{x}}{{T}_{l}-{T}_{x}},$$

Combing the two parts together, we define an index *G*(*a*) here as:

15 $$G(a)=\frac{({T}_{g}-{T}_{0}){{T}_{l}}^{2}}{{T}_{g}{({T}_{l}-{T}_{0})}^{2}}{(\frac{{T}_{x}}{{T}_{l}-{T}_{x}})}^{a},$$

The first item on the right hand side of the above equation is the contribution of the Arrhenius-VFT crossover and the second one with the index *a* is the contribution of the critical temperature. *a* is determined as given in the “Results and discussions” section.

### Data availability

All data analyzed during this study are included in this published article.
